# Electrochemically Driven Swinging of a Nitrobenzyl Pendant Arm in a Nickel Scorpionand Complex

**DOI:** 10.1002/chem.202200462

**Published:** 2022-03-29

**Authors:** Carlo Ciarrocchi, Luigi Fabbrizzi, Maurizio Licchelli, Angelo Taglietti

**Affiliations:** ^1^ Dipartimento di Chimica Università di Pavia 27100 Pavia Italy

**Keywords:** cyclic voltammetry, intramolecular motions, macrocyclic ligands, molecular devices, Ni(II) and Ni(I) complexes

## Abstract

A radical anion −NO_2_^.−^ is formed upon an electrochemically reversible one‐electron reduction of the square‐planar Ni^II^ complex of *N*‐nitrobenzylcyclam. The −NO_2_^.−^ group goes to occupy an axial position of the metal ion, thus establishing a significant electronic interaction with the metal center. In particular, the ESR spectrum supports the occurrence of an electron transfer from −NO_2_^.−^ to the metal, which therefore presents a significant Ni^I^ character. On re‐oxidation, the nitrobenzyl side chain detaches and the Ni^II^ complex is restored, providing an example of a fully reversible redox driven intramolecular motion.

## Graphical abstract

**An electrochemically** driven intra‐molecular motion on cathodic reduction of the Ni^II^ macrocyclic complex **a**, the electron first goes on the nitro group (**b**), then, following the swinging motion of the nitrobenzyl side chain, it is transferred onto the metal centre, giving rise to an authentic Ni^I^ (d^9^) scorpionate complex (**c**).

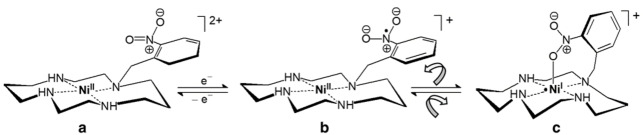

## Introduction

Tetraaza macrocycles (e. g., cyclam) carrying a pendant side‐chain equipped with an additional, potentially coordinating nitrogen atom represent a class of quinquedentate ligands for transition metal ions which couple two distinct properties: (i) the capability of the rigid tetraaza ring to incorporate the metal (e. g., Ni^II^, Cu^II^) giving a kinetically stable species, especially resistant to demetalation; and (ii) the tendency of the flexible pendant arm to further coordinate the metal ion in one of the axial positions of the coordination polyhedron (octahedron, trigonal bipyramid), according to a labile binding mode.^[1]^ The fact that an aggressive tail can bite from the top an immobilised individual (the metal ion) accounts for the current name ligands of this type have been given: *scorpionands*.^[2]^ If the binding group of the side‐chain also displays Brønsted acidic properties, its coordination can be controlled through a pH change.

**Figure 1:**
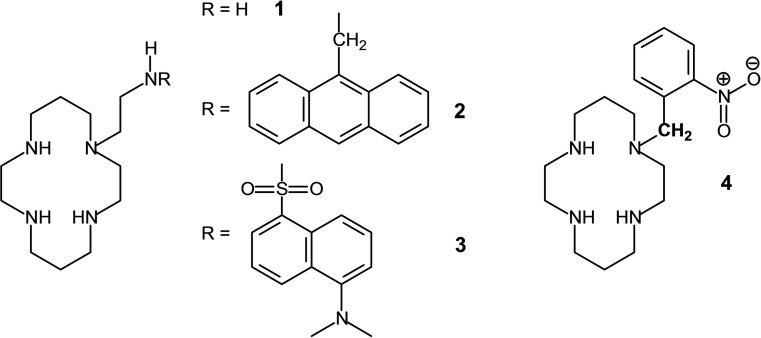
Scorpionands: tetramine macrocycles equipped with a coordinating pendant arm capable to bind from the top an encircled metal ion.

**Figure 2:**
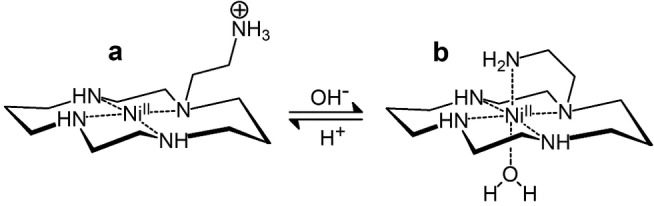
pH controlled swinging of the aminoethyl pendant arm in the [Ni^II^(**1**)]^2+^ scorpionate complex in aqueous solution. Axial binding of the primary amino group induces a change of coordination geometry from square‐planar to octahedral and a spin conversion from high‐ to low‐spin.^[2]^

This is the case of the nickel(II) complex of penta‐amine **1**, in which an ethylamine chain has been appended at an amine nitrogen atom of cyclam (see formula 1 in Figure 1).^[2]^ Below pH 2.8, the primary amine group of the pendant arm is protonated and, due to electrostatic repulsion, lies far away from the metal ion giving rise to a square planar, low‐spin, yellow complex [Ni^II^(LH)]^3+^. Above pH 2.8, the NH_3_^+^ group deprotonates and the −NH_2_ fragment goes to bind the metal. An octahedral high‐spin [Ni^II^(L)H_2_O]^2+^ species forms, according to the equilibrium illustrated in Figure 2, whose p*K*_A_ is 2.8.

The remarkable higher acidity of the −NH_2_ group of the pendant arm with respect to chosen reference systems (ethylamine p*K*_A_ 10.71, ethylenediamine p*K*_A1_ 6.87)^[3]^ cannot be simply ascribed to the energy gain associated to the metal coordination by the pendant arm, an energy presumably not too different from that associated to the addition of a fifth molecule of ammonia to Ni^2+^ (log *K*_5_=0.8). This leaves room to a determining additional term, related to the facilitated binding of a donor group linked by a short aliphatic chain to an already metal bound donor atom (a *chelate effect*).^[4]^ Chelate effects corresponding to 3–4 log units are typically observed in coordination chemistry of multidentate ligands.

In the nickel(II) complex of scorpionand **2** (p*K*_A_=3.37), whose pendant amine group has been equipped with a light‐emitting subunit (9‐anthracenyl), the position of the side‐chain is signalled by fluorescent emission, whether bound (fluorescence OFF) or not bound (fluorescence ON).^[5]^ A similar ON‐OFF behaviour was displayed by the nickel(II) complex with scorpionand **3** (light emitting subunit: dansylamide), whose coordinating group, a sulfonamide, deprotonates and binds the metal with a p*K*_A_=4.65.^[6]^

**Figure 3:**
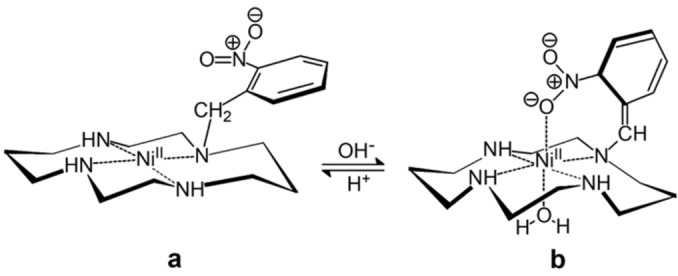
The deprotonation of ‐CH_2_‐ group of the 2‐nitrobenzyl pendant arm of the scorpionate complex [Ni^II^(**4**)]^2+^ (form **a**) promotes the coordination of the nitronate group to the nickel(II) centre (form **b**), and causes a change of the coordination geometry from square‐planar to octahedral and a spin conversion from high‐ to low‐spin.^[7]^

Scorpionands containing donor atoms different from nitrogen are more rare. A special case refers to the macrocycle **4**, *N*‐nitrobenzylcyclam.^[7]^ The nitro group itself does not show any coordinating tendency and the [Ni^II^(**4**)]^2+^ complex (form **a** in Figure 3) exhibits a square planar coordination geometry (low‐spin state). In basic solutions, the −CH_2_− fragment linking the 2‐nitrobenzene subunit to a nitrogen atom of cyclam deprotonates to give the nitronate form, which goes to coordinate apically the Ni^II^ centre. A high‐spin octahedral complex forms, the other apical position being occupied by a water molecule (form **b** in Figure 3). In particular, the [Ni^II^(**4**)]^2+^ complex shows a p*K*_A_ of 10.71, a value remarkably lower than that of the reference system 4‐nitrotoluene, p*K*_A_=20.6, which confirms the central role of the chelate effect on the stabilisation of scorpionate complexes.^[7]^

At this stage, we wanted to verify whether: (i) a negative charge on the nitro group could be generated also by direct electrochemical reduction, and (ii) the electrogenerated −NO_2_^.−^ radical group could bind the metal centre according to a scorpionate mode. We report here a study on the electrochemical behaviour of the [Ni^II^(**4**)]^2+^ complex in MeCN. ESR studies on the solution stable [Ni(**4**)]^+^ species, obtained through one‐electron reduction in a controlled potential electrolysis experiment, supported the occurrence of an electron transfer from −NO_2_^.−^ to the metal, which therefore presents a significant Ni^I^ character.

## Experimental Section

**Materials and instrumental methods**: Unless otherwise stated, all reagents and solvents were supplied by Sigma‐Aldrich and used as received. Nitrobenzylcyclam (**4**, 1‐(2‐nitrobenzyl)‐1,4,8,11‐tetra‐azacyclotetradecane) was synthesised through a modification of a previously reported procedure,^[7]^ as described in Supporting Information. Mass spectra were acquired on a Thermo‐Finnigan ion‐trap LCQ Advantage Max instrument equipped with an ESI source. ^1^H NMR spectra were obtained on a Bruker Avance 400 spectrometer. IR spectra were recorded on a Thermo Fisher Nicolet iS20 equipped with an ATR accessory. Anhydrous MeCN (99.8 %) and [Bu_4_N]ClO_4_ (>98 %) for electrochemical studies were purchased from Sigma‐Aldrich and used as received.

**Electrochemical measurements**: Cyclic voltammetry (CV), differential pulse voltammetry (DPV) and controlled potential coulometry (CPC) were performed with a BAS 100B potentiostat/galvanostat, under the control of a PC, with dedicated software. CV and DPV experiments were carried out in a three‐electrode cell on anhydrous MeCN (99,8 %) solutions of the investigated compounds (2.5×10^−3^−5×10^−3^ mol L^−1^; 0.1 mol L^−1^ [Bu_4_N]ClO_4_ as inert electrolyte). A platinum electrode was used as a working electrode, a platinum wire as an auxiliary electrode, and Ag/Ag^+^ as the pseudo‐reference electrode made by a clean silver wire dipped into a MeCN filling solution 10^−2^ M in AgNO_3_ and 0.1 M in [Bu_4_N]ClO_4_. In each experiment ferrocene was added as an internal standard. CPC experiments were carried out on an MeCN solution of complex [Ni(**4**)]^2+^ using a platinum gauze as a working electrode. The counter‐electrode compartment was separated from the working compartment by a reverse U‐shaped bridge, filled with MeCN 0.1 M [Bu_4_N]ClO_4_. The reference electrode, a platinum wire dipped in the working cell, was calibrated through CV experiments prior to CPC. Absorption spectra of the solution were measured in the working cell during the electrolysis experiment by using a quartz fibre optic probe (Hellma, 661.500‐QX), connected to a diode‐array spectrophotometer (Cary 8454, Agilent Technologies).

**ESR measurements** were performed with a Bruker EMX‐10/12 (Bruker BioSpin GmbH, Karlsruhe, Germany) operating in the X‐band and equipped with a ER4119HS cavity and temperature control.

## Results and Discussion

**Figure 4:**
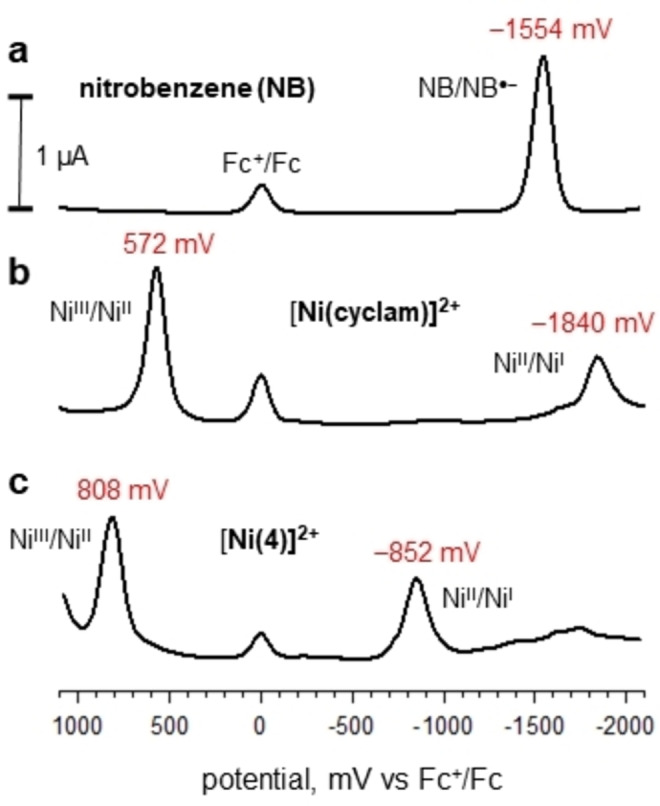
Differential Pulse Voltammetry profiles obtained at platinum microsphere working electrode, in an MeCN solution 0.1 M in Bu_4_NClO_4_, potential scan rate 10 mV s^−1^, T=25 °C: (a) nitrobenzene (NB) (**5**); (b) [Ni^II^(cyclam)]^2+^; (c) [Ni^II^(**4**)]^2+^. Potentials are reported vs. the ferrocenium/ferrocene (Fc^+^/Fc) internal standard. Abscissae have been adjusted to *E*_1/2_(Fc^+^/Fc)=0.00 V.

The redox behaviour of [Ni^II^(nitrobenzylcyclam)]^2+^ in an MeCN solution was electrochemically investigated. For comparative purposes, the redox behaviour of MeCN solutions of nitrobenzene and of [Ni^II^(cyclam)]^2+^ was electrochemically investigated under the same conditions. Figure 4a shows the DPV profile obtained at a platinum working electrode for a solution of nitrobenzene (**5**) in MeCN made 0.1 M in [Bu_4_N]ClO_4_.

**Figure 5:**
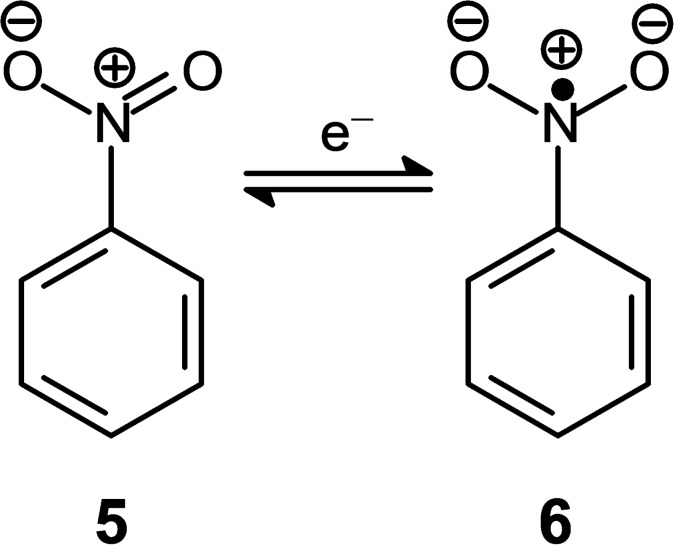
The one‐electron reduction of nitrobenzene to give the radical anion C_6_H_5_NO_2_^•−^ (**6**).

A reversible wave is observed with *E*_1/2_=−1.55 V, corresponding to the formation of the radical anion C_6_H_5_NO_2_^.−^ (**6**), according to the one‐electron reduction process illustrated in Figure 5.

ESR studies have shown that the unpaired electron is mainly localised on the nitrogen atom of the nitro group, which shows a slight tendency to become pyramidal.^[8]^

Coordination of Ni^II^ by plain cyclam promotes a rather rich metal centred redox activity. Figure 4b displays the DPV profile obtained on an MeCN solution of [Ni^II^(cyclam)]^2+^, made 0.1 M in Bu_4_NClO_4_. It is observed that the complex undergoes a reversible one‐electron oxidation to Ni^III^ with *E*_1/2_=0.57 V versus Fc^+^/Fc and one‐electron reduction to Ni^I^ with *E*_1/2_=−1.84 V versus Fc^+^/Fc. The authenticity of nickel oxidation states had been determined through ESR studies on an electrolysed frozen MeCN solutions (Ni^III^, d^7^, low‐spin, octahedral; Ni^I^, d^9^, tetragonal).[^9^, ^10^]

Figure 4c illustrates the DPV behaviour of the [Ni^II^(**4**)]^2+^ system. The complex presents an anodic wave at *E*_1/2_=0.81 V. Such a wave can be ascribed to a metal centred oxidation process involving the formation of a Ni^III^ complex. Noticeably, the Ni^II^‐to−Ni^III^ oxidation process for the *N*‐nitrobenzyl cyclam complex takes place at a distinctly more positive potential than that observed for the cyclam complex. According to a traditional Crystal Field perspective, on oxidation of Ni^II^ to Ni^III^ in a tetramine complex of square planar geometry one electron is abstracted from the highest energy orbital, that is, d_xy_. Replacement of a secondary amine nitrogen atom with a benzylic tertiary nitrogen atom is expected to reduce the strength of the metal ligand interaction. This is confirmed by the values of the Ni^II^−N distances observed in the crystalline square‐planar low‐spin complex salt [Ni^II^(**4**)](CF_3_SO_4_)_2_:^[7]^ Ni^II^−N_sec_=1.93 Å, 1.94 Å, 1.93 Å; Ni−N_tert_=1.99 Å. This lowers the energy of the d_xy_ level, thus making the electron removal more difficult. However, it is the reduction behaviour that shows the more significant differences. In particular, a first reduction wave is observed at a *E*_1/2_=−0.85 V, that is, 1 V less negative than observed for [Ni^II^(cyclam)]^2+^.

**Figure 6:**
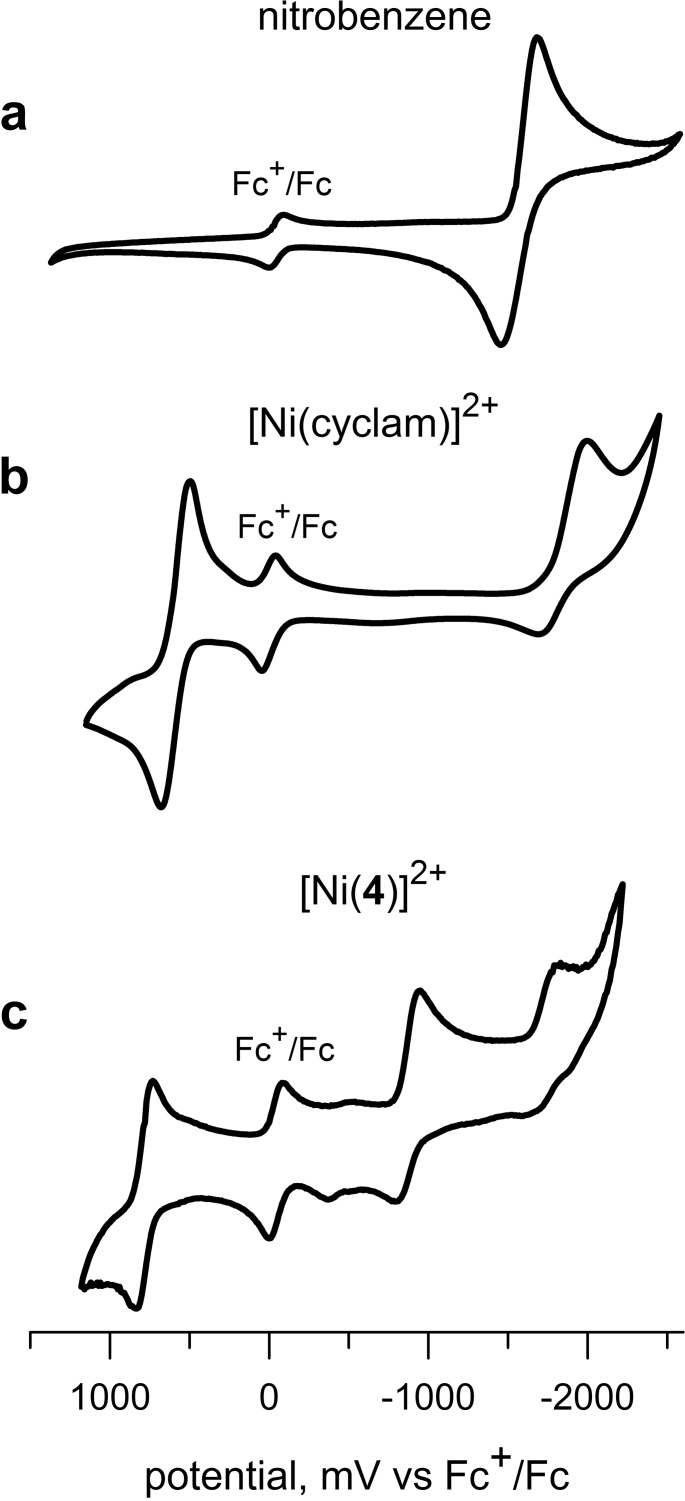
Cyclic voltammetry profiles obtained at platinum microsphere working electrode, in an MeCN solution 0.1 M in Bu_4_NClO_4_, potential scan rate 200 mV s^−1^, T=25 °C: (a) nitrobenzene, **5**; (b) [Ni^II^(cyclam)]^2+^; (c) [Ni^II^(**4**)]^2+^. Potentials are reported vs. the ferrocenium/ferrocene (Fc^+^/Fc) internal standard. Abscissae adjusted to *E*_1/2_(Fc^+^/Fc)=0.00 V.

Figure 6 shows the corresponding cyclic voltammetry profiles taken at platinum working electrode at a potential scan rate of 100 mV s^−1^.

In all cases quasi‐reversible waves were observed with *E*_1/2_ values corresponding to those observed in DPV studies. Small waves in the CV profile of [Ni(**4**)]^2+^ (around −400/−500 mV) should be ascribed to impurities and are not observed in the corresponding DPV profile (Figure 4c). Noticeably, an irreversible wave was observed at ca. −1800 mV versus Fc^+^/Fc, probably corresponding to a second reduction of the [Ni^II^(**4**)]^2+^ complex, followed by decomposition. The occurrence of such a phenomenon was confirmed by control potential electrolysis experiments (see below).

The electrochemical reversibility of the processes profiles shown in Figure 4 was tested by plotting the current intensity versus the square root of the potential scan rate. A linear behaviour was observed. The peak‐to‐peak difference increased with scan rate excluded the presence of adsorbed species (see Figures S1 and S2 in Supporting Information).

**Figure 7:**
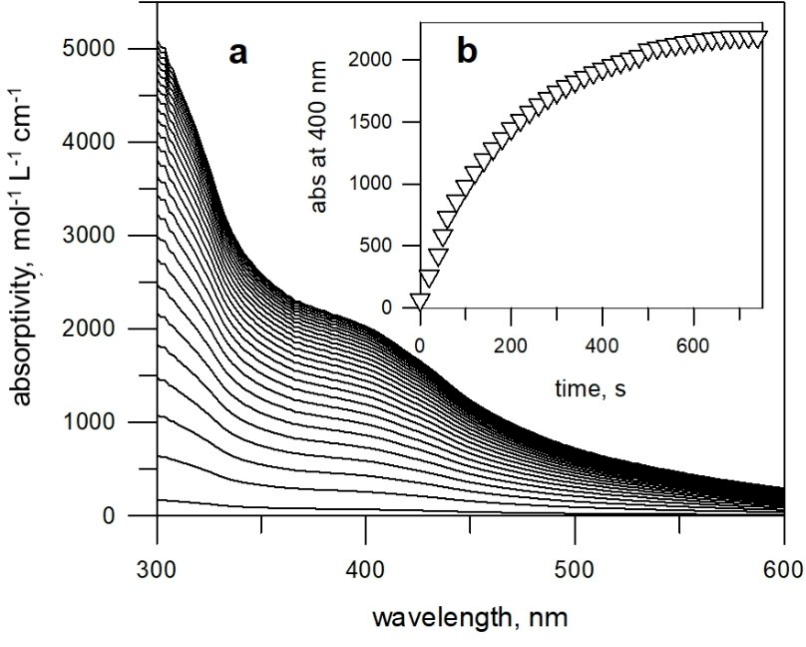
( a) absorption spectra of an MeCN solution 2.22×10^−4^ M in [Ni^II^(**4**)]^2+^, taken over the course of a controlled potential electrolysis (working potential: −1100 mV vs. Fc^+^/Fc); spectra taken every 20 s; (b) molar absorptivities at 400 nm.

The stability of the first reduction product of the [Ni^II^(**4**)]^2+^ complex was spectroscopically investigated. A bulk solution of the reduced form of [Ni^II^(**4**)]^2+^ was prepared by an exhaustive controlled potential electrolysis experiment on an MeCN solution of the [Ni^II^(**4**)](ClO_4_)_2_ complex salt. The potential of the working electrode (a platinum gauze) was set at −1.1 V, that is, 250 mV more negative than the *E*_1/2_ value of the first reduction wave. According to Nernst's equation, a Δ*E* of −236 mV (=59×4) ensures a ratio of the concentrations of Ni^I^ and Ni^II^ species of 1000/1. Moreover, the value of the set potential is well before the potential of the second reduction wave, which excluded any contamination by the second reduction product. The electrolysis experiment was monitored by measuring the absorption spectra with an optical fibre dipped in the solution and connected to a diode array spectrophotometer. The family of recorded spectra is shown in Figure 7.

During the experiment, the pale yellow solution progressively took a bright yellow colour, while an intense absorption band developed with a shoulder at 385 nm, which reached a limiting value of ϵ=2.505 M^−1^ cm^−1^ (at 385 nm). It is suggested that such an intense band results from a charge transfer transition from the −NO_2_^.−^ radical to the Ni^II^ centre. On coulometry, the passage of 1±0.1 electron per molecule of complex was determined. Bulk electrolysis was carried out also at 2.0 V, that is, at a potential 200 mV more negative than the *E*_1/2_ of the second reduction wave. However, during the experiment the current did not decrease, but kept a constant value, indicating decomposition.

**Figure 8:**
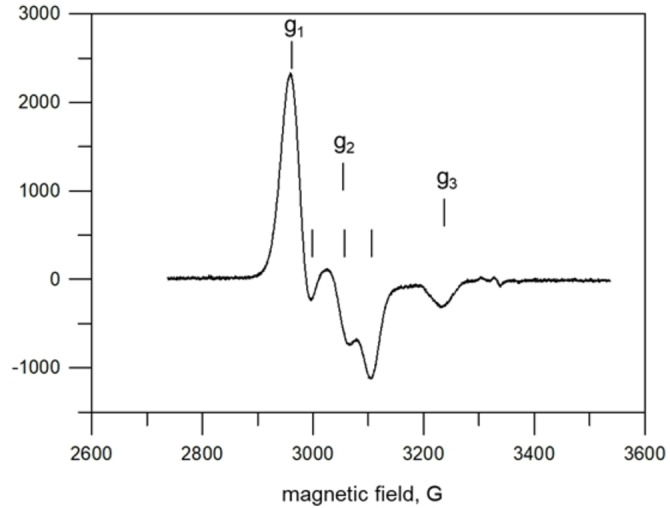
ESR spectrum of an MeCN solution of [Ni^II^(**4**)]^2+^, cathodically reduced at −1.1 V vs. Fc^+^/Fc and frozen at 113 K.

The nature of the reduced species [Ni(**4**)]^+^ was investigated by carrying out ESR investigations on the electrolysed solution. Figure 8 displays the ESR spectrum of the electrolysed solution, 6.07×10^−4^ M in the metal complex, frozen at 113 K.

The spectrum shows a rhombic symmetry, with g_1_=2.260, g_2_=2.198, g_3_=2.072. The g_2_ feature exhibits a hyperfine structure, with a coupling constant: A_2_=50 G. The spectrum is completely different from that expect for an uncoordinated nitrobenzene radical anion.^[8]^ The large low‐field shift of g values indicates a rather intense interaction of the nitro group with the metal centre. In particular, the pattern of g values strongly supports the occurrence of an electron transfer to the metal, which therefore presents a significant Ni^I^ character. ESR spectra with similar g patterns have been previously reported for a variety of Ni^I^ macrocyclic complexes.[^11^, ^12^, ^13^]

**Figure 9:**
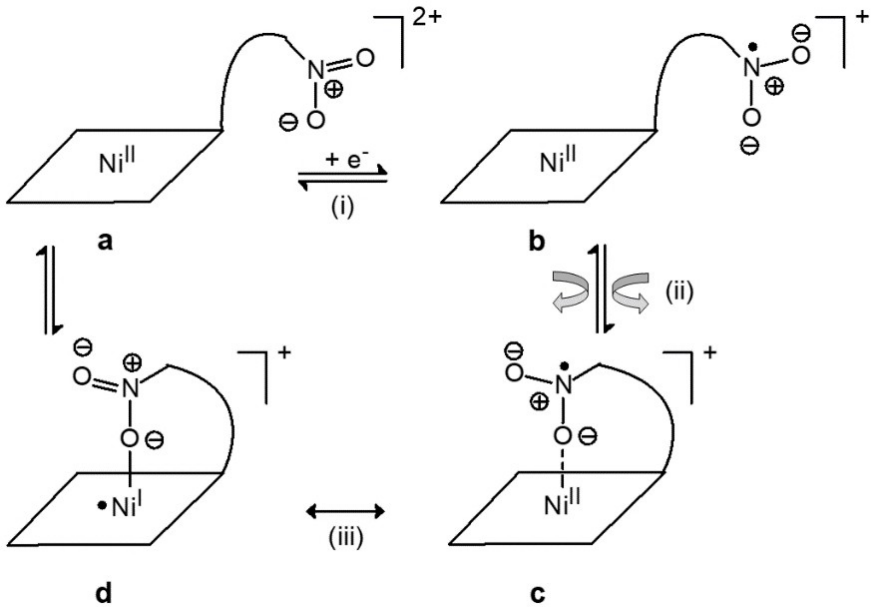
Geometrical aspects of the one‐electron reduction of the [Ni^II^(**4**)]^2+^ complex (**a**). The process involves: (i) the electron transfer from the electrode to the nitrogen atom of the nitro group, to give (**b**); (ii) the movement of the pendant arm to coordinate by an oxygen atom of the −NO_2_^.−^ group the metal centre, to give **c**; (iii) the electron transfer from −NO_2_^.−^ to a metal centred orbital of Ni^II^ (mostly $d_{x^{2}}{\;}_{-}{\;}_{y^{2}}$ in character), to give the Ni^I^ complex **d**.

The reduction process at −0.85 V versus Fc^+^/Fc can be ideally dissected into three successive steps (not necessarily in a chronological sequence), as illustrated in Figure 9: (i) one electron is uptaken from the electrode by the nitro group of the complex species **a**, to generate **b**; (ii) an ample motion of the pendant nitrobenzyl fragment takes place, which may involve the rotation of the nitrobenzene ring, to put an oxygen atom of the −NO_2_^.−^ group at a bonding distance with the metal centre, to give form **c**. Then, an electron transfer occurs from the −NO_2_^.−^ radical fragment to the metal, which is reduced to the Ni^I^ oxidation state (**d**).

Notice that the final reduction product is represented in Figure 9 as a resonance hybrid between the Ni^II^ complex **c**, interacting with the −NO_2_^.−^ radical anion and the Ni^I^ species **d**. Actually, while the rhombic three‐g feature suggests a predominant Ni^I^ character, the triplet hyperfine structure of g_2_ indicates that the unpaired electron still interacts with the nitro group.

The process is fully reversible, as shown by the fact that the reverse controlled potential electrolysis experiment makes the bright yellow colour vanish, while the EPR signal disappears (due to the formation of the ESR silent [Ni^II^(**4**)]^2+^ species).

The effect of −NO_2_^.−^ coordination on the stabilisation of the Ni^I^ state can be evaluated by considering the difference of the *E*_1/2_ potentials: Δ*E*_1/2_=*E*_1/2_(Ni^II/I^(**4**)]^2+/+^)−*E*_1/2_(Ni^II/I^(cyclam)]^2+/+^)=1.0 V. On assuming that Δ*E*_1/2_=Δ*E*°, the corresponding Δ*G*° can be calculated: Δ*G*°=−*n*FΔ*E*°=−23.06 kcal mol^−1^, which is the free energy change associated to the following equilibrium:

(1) $$\mathrm{Ni}{}^{\mathrm{II}}\left(4\right){}^{2+}+\mathrm{Ni}{}^{I}\left(\mathrm{cyclam}\right){}^{+}\overset{\leftarrow }{\to }\mathrm{Ni}{}^{I}\left(4\right){}^{2+}+\mathrm{Ni}{}^{\mathrm{II}}\left(\mathrm{cyclam}\right){}^{2+}$$

It follows that the constant *K* of Eq. (1) is 10^17^, which highlights the dramatic effect of axial coordination of a pendant arm equipped with −NO_2_^.−^ on the formation of the Ni^I^ state.

It has been mentioned that, in complexes with pH‐sensitive scorpionands, metal chelation modifies the distinctive property of the pendant arm, by increasing significantly its Brønsted acidity. In the case of electrochemically active scorpionands the distinctive property under investigation is the tendency of the nitrobenzene group to be reduced. Indeed, when linked to the Ni^II^(cyclam) subunit, the nitrobenzene fragment is reduced at a potential of −0.83 V, a value much less negative than that of the isolated nitrobenzene fragment (−1.56 V). Ease of reduction has to be ascribed to the fact that the electron released by the electrode is in most part transferred to nickel and that the achievement of the Ni^I^ state is facilitated by the pendant arm coordination.

Crown ethers equipped with a nitrobenzyl functionality have been previously reported by Gokel (belonging to the family of lariat ethers, that is, polioaxa macrocycles with a pendant arm).^[14]^ Titration with an alkali metal ion (e. g., Na^+^) induced a progressive and moderate anodic shift of the reduction wave of the nitrobenzene fragment, in CV experiments carried out in MeCN solvent. The effect was merely electrostatic and, in contrast to what observed in this work, ESR studies showed that no electron transfer took place from the reduced nitrobenzene fragment to the metal.^[15]^ It should be noted that the electron transfer to the metal centre in the investigated complex may be also favoured by the 2+ charge of nickel, compared to the monopositive charge of Na^+^.

## Conclusions

This work has demonstrated (i) that the −NO_2_^.−^ radical group can coordinate a metal centre in a scorpionand complex, and (ii) a definite and reversible intramolecular motion (the swinging of the pendant arm) can be promoted by a variation of the redox potential. The first classical example of redox promoted molecular movements refer to catenanes and rotaxanes designed by Stoddart (in which the redox change modified the π‐donor/acceptor properties of given fragments inserted in the axle or in the wheel)^[16]^ and by Sauvage (in which the redox change involved a modification of the coordination geometry connected to the Cu^I^/Cu^II^ couple).^[17]^ These examples represented the first examples of molecular machines, and opened a new theme, which was highly investigated during the following decades,[^18^, ^19^, ^20^, ^21^, ^22^, ^23^, ^24^] and culminated in the award of the Nobel Prize to Sauvage, Stoddart and Feringa in 2016.^[25]^ Notice that in rotaxanes and catenanes the stationary and the mobile parts are held together by non‐covalent interactions. In scorpionands **1**–**4**, the stationary unit (the tetramine macrocycle) and the mobile moiety (the pendant arm) are covalently linked. While pH‐controlled swinging of the side‐chain in scorpionate complexes of type **1**–**3** is known since 1977, present work has demonstrated that the pendant arm can be made oscillate at will by a change of the redox potential.

Among scorpionand complexes, [Ni^II^(**4**)]^+^ is unique, in that it is able to convert into a definite motion the energy of either chemical (acid‐base) or electrochemical nature.^[26]^ Moreover, it offers an unprecedented versatility as one (i) can equip the pendant arm with redox active fragments of various nature and (ii) can choose among a variety of transition metals, all displaying different redox activity and geometrical preferences. Studies along these lines are underway in this Laboratory.

## Conflict of interest

The authors declare no conflict of interest.

1

## Supporting information

As a service to our authors and readers, this journal provides supporting information supplied by the authors. Such materials are peer reviewed and may be re‐organized for online delivery, but are not copy‐edited or typeset. Technical support issues arising from supporting information (other than missing files) should be addressed to the authors.

Supporting Information

## Data Availability

The data that support the findings of this study are available in the supplementary material of this article.
