# Supplementary material for: Electrochemically Driven Swinging of a Nitrobenzyl Pendant Arm in a Nickel Scorpionand Complex
Source: Chemistry. 2022 Mar 29;28(26):e202200462. doi: 10.1002/chem.202200462 (PMC9311437; doi:10.1002/chem.202200462)
Supplement: Supplementary file 1 — Supporting Information [file CHEM-28-0-s001.pdf]

Chemistry–A European Journal

Supporting Information

Electrochemically Driven Swinging of a Nitrobenzyl Pendant Arm in a Nickel Scorpionand Complex

Carlo Ciarrocchi, Luigi Fabbrizzi,* Maurizio Licchelli, and Angelo Taglietti

Synthesis

Cyclam,[a]1,4,8-tris(tertbutoxycarbonyl)-1,4,8,11-tetraazacyclotetradecane (trisBOCcyclam),[b] and 2-nitrobenzyl iodide[c] were prepared according to literature methods. Complex [Ni(**4**)]$^{2+}$ was prepared according to a modification of the reported procedure, as depicted in Scheme S1.

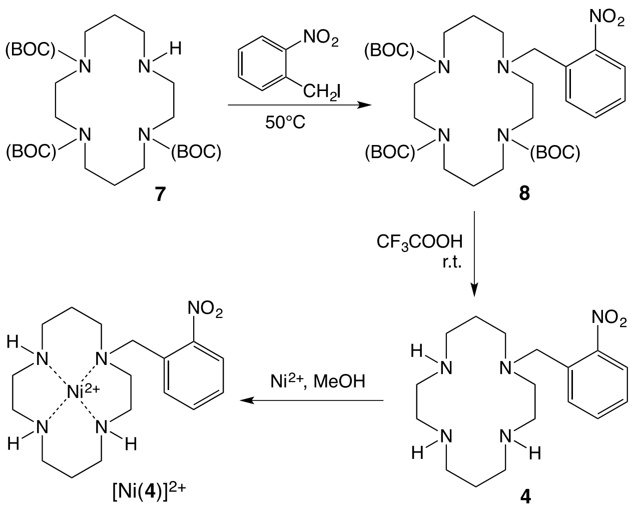

Scheme S1. Synthesis of the scorpionand complex.

1-(2-nitrobenzyl)-4,8,11-tris-*t*-butoxycarbonyl-1,4,8,11-tetraaza-cyclotetradecane, **8**. A solution of 2-nitrobenzyl iodide (105 mg, 0.4 mmol) in anhydrous acetonitrile (99,8%, 15 mL) was added dropwise to a solution of tris-*BOC*-Cyclam, **7** (200 mg, 0.4 mmol)[b] in anhydrous MeCN (30 ml), containing K_2CO_3 (110 mg, 0.8 mmol) and the resulting mixture was stirred at 50°C for 20h. K_2CO_3 was filtered and the solvent was removed from the reaction mixture by a rotary evaporator to give an oily product. The oil was purified by liquid cromatography (silica gel, EtOAc-hexane, 1:2) to give **8**. Yield: 70%. MS (ESI): m/z (%) 636.4 (100) [**8**+H]$^+$. ^{1}H NMR (400 MHz, $CDCl_3$): δ=7.81 (m, 1H), 7.54 (m, 2H), 7.41 (m, 1H), 3.80 (s, 2H), 3.5-3.1 (m, 12H) 2.7-2.2 (m, 4H) 2.0-1.6 (m, 4H), 1.5-1.2 (m, 27H).
IR (ATR): ν~ = 2973, 2934, 1683 (C=O), 1526 (N-O), 1463, 1410, 1364 (N-O), 1242, 1151 cm^{-1}.

1-(2-nitrobenzyl)-1,4,8,11-tetraaza-cyclotetradecane, **4.** Compound **8** (140 mg, 0,22 mmol) was dissolved in dichloromethane (9 mL) and CF_3COOH (0,6 mL) was added. The resulting mixture was stirred at room temperature for 24 h, then it was extracted with water (3 x 5 mL). The aqueous phase was treated with excess NaOH until it became turbid and extracted with dichloromethane (4 x 7 mL). The organic phase was desiccated over anhydrous sodium sulfate. After filtering the desiccated salt, the solvent was completely removed by a rotary evaporator and scorpionand **4** was obtained as an oily product, which slowly became solid after extended treatment *in vacuo*. Yield: 80%. MS (ESI): m/z (%) 336.5 (100) [**4**+H]$^+$. ^{1}H NMR (400 MHz, $CDCl_3$): δ= 7.83 (d, 1H), 7.80 (d, 1H), 7.55 (false t, 1H), 7.38 (false t, 1H), 3.81 (s, 2H), 2.5-2.8 (m, 16H), 1.7 (m, 4H). IR (ATR): ν~ = 3273, 3190 (N-H), 2920, 2872, 2800, 1522 (N-O), 1460, 1341 (N-O), 1120 cm^{-1}.

The nickel(II) complex [Ni(4)](ClO_4)$_2$ was obtained on adding a methanolic solution of $Ni(ClO_4)_2 \cdot 6H_2O$ (47.5 mg, 0.13 mmol) to a methanolic solution of **4** (40 mg, 0.12 mmol). The

mixture was refluxed for 2 h, and an orange-yellow precipitate formed. The solid [Ni(**4**)](ClO_4)$_2$ was filtered, washed with cold methanol and dried (70% yield). MS (ESI) m/z (%): 196.7 (100) [Ni(**4**)]$^{2+}$, 392.1 (5) [Ni(**4**)-H]$^+$, 492.1 (5) [Ni(**4**)+ClO_4]$^+$. IR (ATR): ν~ = 3200 (N-H), 2939, 2882, 1525 (N-O), 1471, 1347 (N-O), 1062 (ClO_4^-) cm^{-1}.

[a] E. K. Barefield, F. Wagner, A. W. Herlinger, A. R. Dahl, *Inorg. Synth.* **1975**, *16*, 220–225.

[b] L. Fabbrizzi, F. Foti, M. Licchelli, P. M. Maccarini, D. Sacchi, M. Zema, *Chem. Eur. J.* **2002**, *8*, 4965–4972.

[c] M. Alajarín, A. Pastor, R.-Á. Orenes, J. W. Steed, R. Arakawa, *Chem. Eur. J.* **2004**, *10*, 1383–1397.

Cyclic Voltammetry at varying potential scan rate

The electrochemical reversibility of the investigated redox processes was investigated by carrying out cyclic voltammetry experiments at increasing potential scan rates. According to the Randles- Ševčík equation, in electrochemically reversible electron transfer processes, controlled by the diffusion of the redox species, the peak current is expected to increase linearly with the square root of the potential scan rate.[d] A linear dependence of the peak current intensity from the square root of the potential scan rate was observed for the processes investigated in this work, as illustrated in the following Figures. The increase of the peak-to-peak difference increased with scan rate excluded the presence of adsorbed species.[e]

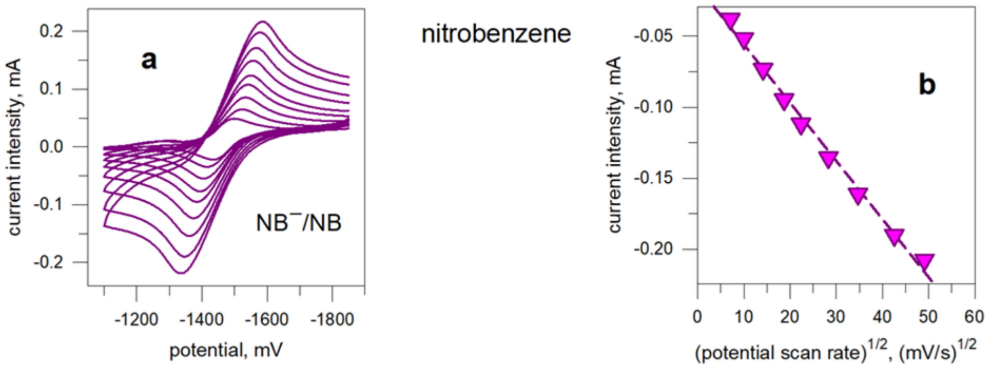

Figure S1. (a) Cyclic voltammetry profiles of the NB$^{\bullet-}$/NB (NB = nitrobenzene) taken on a platinum working electrode at varying potential scan rates (mV s^{-1}): 50, 100, 200, 350, 500, 800, 1200, 1800, 2400); (b) linear dependence of the current intensity of the cathodic peak vs the square root of the potential scan rate.

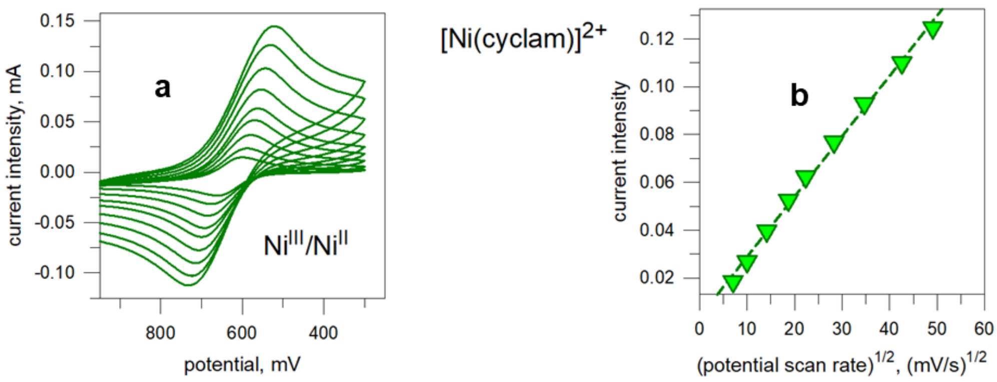

Figure S2. (a) Cyclic voltammetry profiles of the Ni^{III}/Ni^{II} redox change for the $[Ni(cyclam)]^{2+}$ complex taken on a platinum working electrode at varying potential scan rates (mV s^{-1}): 50, 100, 200, 350, 500, 800, 1200, 1800, 2400); (b) linear dependence of the current intensity of the cathodic peak vs the square root of the potential scan rate.

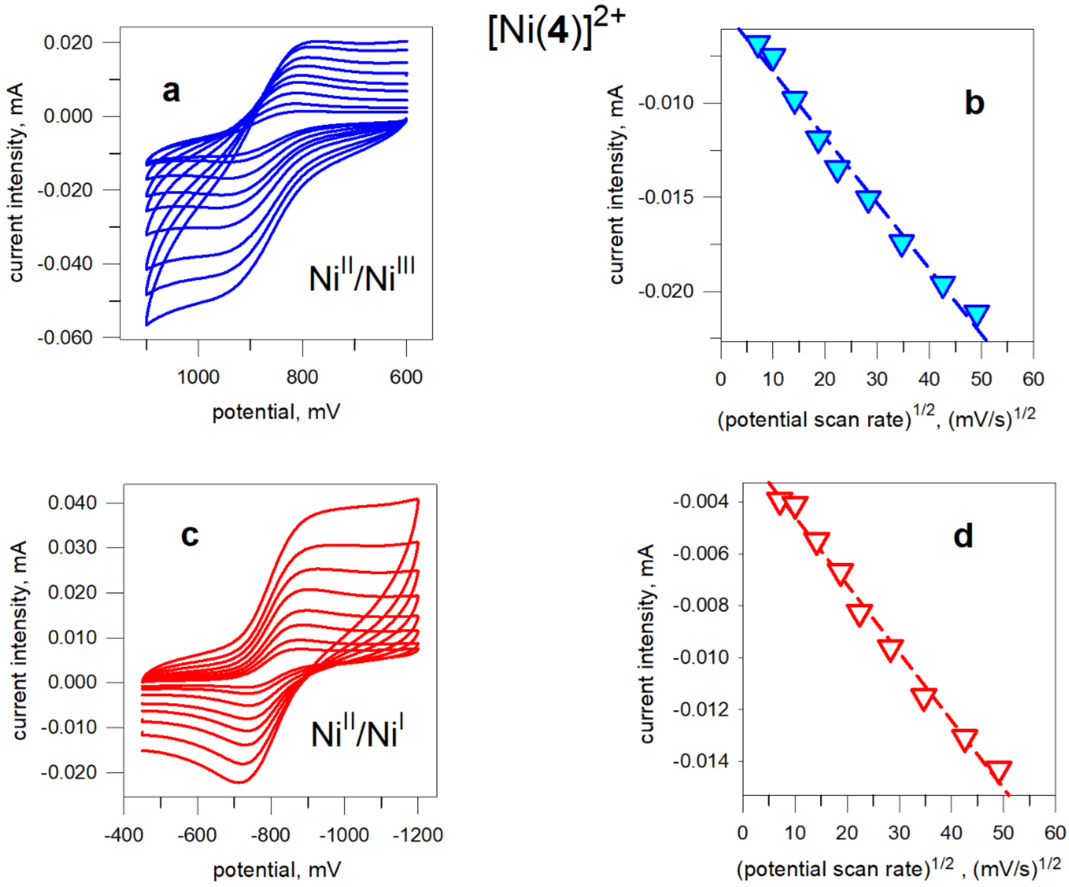

Figure S5. Cyclic voltammetry profiles taken on a platinum working electrode at varying potential scan rates (mV s^{-1}): 50, 100, 200, 350, 500, 800, 1200, 1800, 2400: (a) Ni^{II}/Ni^{III} redox change; (b) linear dependence of the current intensity of the cathodic peak vs the square root of the potential scan rate; (c) Ni^{II}/Ni^{I} redox change; (d) linear dependence of the current intensity of the anodic peak vs the square root of the potential scan rate.

(d) R. S. Nicholson, I. Shain, Theory of Stationary Electrode Polarography. Single Scan and Cyclic Methods Applied to Reversible, Irreversible, and Kinetic Systems, *Anal. Chem.* **1964**, *36*, 706–723

(e) N. Elgrishi, K. J. Rountree, B. D. McCarthy, E. S. Rountree, T. T. Eisenhart, J. L. Dempsey, A Practical Beginner's Guide to Cyclic Voltammetry, *J. Chem. Educ.* **2018**, *95*, 197–206.
